# Age-Related Seroprevalence of Antibodies Against AAV-LK03 in a UK Population Cohort

**DOI:** 10.1089/hum.2018.098

**Published:** 2019-01-24

**Authors:** Dany P. Perocheau, Sharon C. Cunningham, Juhee Lee, Juan Antinao Diaz, Simon N. Waddington, Kimberly Gilmour, Simon Eaglestone, Leszek Lisowski, Adrian J. Thrasher, Ian E. Alexander, Paul Gissen, Julien Baruteau

**Affiliations:** ^1^ Genetics and Genomic Medicine Programme, Great Ormond Street Institute of Child Health, University College London, London, United Kingdom.; ^2^ Gene Transfer Technology Group, Institute for Women's Health, University College London, London, United Kingdom.; ^3^ Gene Therapy Research Unit, Children's Medical Research Institute, Faculty of Medicine and Health, University of Sydney and Sydney Children's Hospital Network, Westmead, Australia.; ^4^ Antiviral Gene Therapy Research Unit, Faculty of Health Sciences, University of the Witswatersrand, Johannesburg, South Africa.; ^5^ Clinical Immunology Department, Great Ormond Street Hospital for Children NHS Foundation Trust, London, United Kingdom.; ^6^ Translational Research Office, University College London, London, United Kingdom.; ^7^ Translational Vectorology Group, Children's Medical Research Institute, Faculty of Medicine and Health, University of Sydney, Westmead, Australia.; ^8^ Military Institute of Hygiene and Epidemiology, The Biological Threats Identification and Countermeasure Centre, Puławy, Poland.; ^9^ Infection, Immunity and Inflammation Programme, Great Ormond Street Institute of Child Health, University College London, London, United Kingdom.; ^10^ Discipline of Child and Adolescent Health, Sydney Medical School, Faculty of Medicine and Health, University of Sydney, Westmead, Australia.; ^11^ MRC Laboratory for Molecular Biology, University College London, London, United Kingdom.; ^12^ Metabolic Medicine Department, Great Ormond Street Hospital for Children NHS Foundation Trust, London, United Kingdom.

**Keywords:** adeno-associated virus, AAV, gene therapy, liver, neutralizing antibodies, seroprevalence, AAV-LK03

## Abstract

Recombinant adeno-associated virus (rAAV) vectors are a promising platform for *in vivo* gene therapy. The presence of neutralizing antibodies (Nab) against AAV capsids decreases cell transduction efficiency and is a common exclusion criterion for participation in clinical trials. Novel engineered capsids are being generated to improve gene delivery to the target cells and facilitate success of clinical trials; however, the prevalence of antibodies against such capsids remains largely unknown. We therefore assessed the seroprevalence of antibodies against a novel synthetic liver-tropic capsid AAV-LK03. We measured seroprevalence of immunoglobulin (Ig)G (i.e., neutralizing and nonneutralizing) antibodies and Nab to AAV-LK03 in a cohort of 323 UK patients (including 260 pediatric) and 52 juvenile rhesus macaques. We also performed comparative analysis of seroprevalence of Nab against wild-type AAV8 and AAV3B capsids. Overall IgG seroprevalence for AAV-LK03 was 39% in human samples. The titer increased with age. Prevalence of Nab was 23%, 35%, and 18% for AAV-LK03, AAV3B, and AAV8, respectively, with the lowest seroprevalence between 3 and 17 years of age for all serotypes. Presence of Nab against AAV-LK03 decreased from 36% in the youngest cohort (birth to 6 months) to 7% in older primary school-age children (9–11 years) and then progressively increased to 54% in late adulthood. Cross-reactivity between serotypes was >60%. Nab seroprevalence in macaques was 62%, 85%, and 40% for AAV-LK03, AAV3B, and AAV8, respectively. When planning for AAV gene therapy clinical trials, knowing the seropositivity of the target population is critical. In the population studied, AAV seroprevalence for AAV serotypes tested was low. However, high cross-reactivity between AAV serotypes remains a barrier for re-injection. Shifts in Nab seroprevalence during the first decade need to be confirmed by longitudinal studies. This possibility suggests that pediatric patients could respond differently to AAV therapy according to age. If late childhood is an ideal age window, intervention at an early age when maternal Nab levels are high may be challenging. Nab-positive children excluded from trials could be rescreened for eligibility at regular intervals because this status may change.

## Introduction

Over the last decade, recombinant adeno-associated virus (rAAV) vectors have shown increasing promise as a platform for *in vivo* gene therapy. More than 200 phase I to phase III clinical trials using AAV vectors have been performed worldwide^1^ and successful results have been achieved in inherited diseases, particularly those affecting the liver,^2–6^ the eye,^7–10^ and the central nervous system.^11–13^ Glybera^®^ (Uniqure, Amsterdam, the Netherlands) and Luxturna^®^ (Spark Therapeutics, Philadelphia, PA) were the first AAV gene therapy products to receive market authorization by the European Medicine Agency^14^ and the Food and Drug Administration^15^ in 2012 and December 2017, respectively. To date, recombinant AAV vectors exploiting both wild-type and engineered capsids have shown good safety profiles in clinical trials.^16,17^

The presence of antibodies with neutralizing effect (*i.e*., preventing target cell transduction) has been recognized as a major limiting factor for gene delivery *in vivo*^18^ even at low titers such as 1 in 5 (1:5) dilution.^19–21^ Therefore, the presence of neutralizing antibodies (Nab) against the vector capsid is widely considered to be an exclusion criterion for recruitment into clinical trials.^22^ Since the seroprevalence against wild-type AAV in humans varies from 40% for AAV8 to 70% for AAV1 and AAV2,^23^ this substantially reduces the proportion of the population that could benefit from AAV-based therapeutics. Factors such as age, geographic location, and capsid species-of-origin influence seroprevalence rates.^24,25^ Cross-reactivity between serotypes is commonly >50%.^26^

Although most clinical successes to date have been obtained with wild-type capsids, great interest exists in the gene therapy field for developing engineered capsids that could improve transduction of specific organs and cell-types^27^ and evade host immunity.^28,29^ This goal has been achieved either by capsid shuffling^30–32^ or by directed evolution with error-prone PCR.^27,33^ Increased target cell tropism could limit the off-target effects and may also allow a reduction in vector dose, decreasing both demand for a clinical-grade vector for therapy and the potential immune response-mediated toxicity in treated patients.^34^ A recently described engineered capsid AAV-LK03 appears to transduce human hepatocytes much better than AAV8, although results from different labs show some inconsistency.^35,48,49^ The AAV-LK03 *cap* sequence consists of fragments from seven different wild-type serotypes (AAV1, 2, 3B, 4, 6, 8, 9), although AAV3B, a capsid known for its human hepatocyte tropism,^36^ represents 97.7% of the *cap* gene sequence and 98.9% of the amino acid sequence.^35^ A first-in-human trial (NCT03003533) using AAV-LK03 for hemophilia A sponsored by Spark Therapeutics is in progress.^37^

Little is known about human immunoreactivity to engineered capsids. Although seroprevalence of antibodies against AAV-LK03 capsid has been reported to be in a similar range to its close parental wild-type capsid AAV3B in a Chinese population (91% and 89%, respectively),^38^ Nab titers against the synthetic capsid are thought to likely be lower than those against the AAV-3B in humans.^39^ There is a particular lack of epidemiological studies of seroprevalence against wild-type^30^ and engineered^38^ AAV capsids in pediatric populations.^24,25^

Here, we studied the overall IgG (neutralizing and nonneutralizing) and Nab seroprevalences against wild-type capsids AAV3B and AAV8 and engineered capsid AAV-LK03 in UK cohorts of pediatric and adult patients as well as juvenile nonhuman primate serum samples. We showed that Nab seroprevalence in our cohorts was lower than previously described for the three serotypes tested. In addition, we found that Nab seroprevalence decreased during the first decade of life and then increased in adolescence and adulthood. The latter finding was in contrast with IgG seroprevalence, which increased steadily with age.

## Methods

### Samples

Anonymized human serum samples were provided by the Immunology laboratory, Great Ormond Street Hospital for Children NHS Foundation Trust, London, UK, between April 2016 and August 2017 according to the guidelines of the Royal College of Pathologists. To compare seroprevalence rate with a pediatric population, serum samples from juvenile Vietnamese rhesus macaques (*Macaca mulata*) aged ≤18 months old were provided by Envigo Ltd (Huntingdon, UK).

### Vector production

AAV vectors encapsidated with the AAV-LK03 and AAV-3B capsids were provided by Dr. Leszek Lisowski and Dr. Sharon Cunningham from the Translational Vectorology and the Gene Therapy Research Unit, respectively, at the Children's Medical Research Institute, Westmead, Australia. Vector encapsidated with the AAV8 capsid was produced at the Gene Transfer Technology Unit, UCL Institute for Women's Health, London. The recombinant AAV genomes contained AAV2 inverted terminal repeats flanking a GFP reporter gene under the transcriptional control of the liver-specific hAAT promoter. HEK293T cells were used to package the AAV vectors using a triple transfection protocol as described previously.^40^

Briefly, 72 h post-transfection, cells were scraped and collected in TD buffer (NaCl 140 mM, KCl 5 mM, K_2_HPO_4_ 0.7 mM, MgCl_2_ 3.5 mM, Tris 25 mM adjusted to pH 7.5). Before purification, the cells were treated with five freeze–thaw cycles to lyse the cells and release the viral particles. AAV particles were purified by either double CsCl gradient centrifugation for AAV-LK03 and AAV3B or protein interaction on an AVB sepharose column for AAV8. To remove the glycine and restore the pH, dialysis was performed overnight in 1 × phosphate-buffered saline (PBS) and the purified vector concentrated by ultrafiltration using an Amicon Ultra15_10kDa MWCO (Merck Millipore Corporation, Temecula, CA). Titers were determined by real-time PCR and expressed as vector genomes per milliliter as previously described.^35^

### Anti AAV-LK03 ELISA

For enzyme-linked immunosorbent assay (ELISA), 96-well plates (Corning, CLS3590, Merck, Darmstadt, Germany) were coated with 50 μL of rAAV-LK03 particles at a concentration of 2 × 10^10^ vg/mL in 0.1 M carbonate buffer, pH 9.5 and left to incubate overnight at 4°C. Plates were washed four times with blocking buffer (2% bovine serum albumin in PBS) and subsequently blocked for 2 h at room temperature. Following heat inactivation at 56°C for 30 min, serum to be tested was diluted in blocking buffer at dilutions ranging from 1:30 to 1:65,610. Fifty microliters of each diluted serum sample was added per well in triplicates and plates were incubated for 1 h at 37°C. Plates were then washed four times with wash buffer (0.05% Tween 20 in PBS). Wells not coated with rAAV particles but blocked and subsequently incubated with the serially diluted serum served as negative controls of background signal. Each well was then incubated with a 1:2,000 dilution of HRP-conjugated anti-human IgG (Abcam, Cambridge, UK) in wash buffer for 1 h at room temperature. Finally, plates were washed four times with wash buffer and revealed with tetramethylbenzidine substrate solution (ThermoFisher Scientific, Rockford, IL) for 15 min. The reaction was stopped with a 2 N H_2_SO_4_ solution and the plate was read at 450 nm in a FLUOstar Omega spectrophotometer (BMG Labtech, Ortenberg, Germany) within 30 min of having stopped the reaction. Results were expressed in arbitrary units in optical density (OD). The OD values from background were subtracted from the sample values from each corresponding serum sample. Samples with a signal (OD AAV − OD background) higher than a cutoff point of 0.825, determined as the average signal readings from 20 seronegative samples at a serum titer of 1:2, were considered positive for anti-AAV-LK03 IgG antibody. The ELISA titer was considered to be the highest dilution at which the signal was higher than the cutoff point.

### Neutralizing assay

Human hepatocellular carcinoma HuH-7 cells were seeded at 5 × 10^4^ cells per well on a 48-well plate and left for at least 7 h before transduction. Before use, sera were heat-inactivated at 56°C for 30 min. Precipitate resulting from heat inactivation was removed by centrifugation at 13,000 × *g* for 10 min. On the day of transduction, dilution of serum sample was performed in Dulbecco's modified Eagle's medium (DMEM) (Gibco, Invitrogen, Grand Island, NY) without fetal calf serum (FCS) (JRH, Biosciences, Lenexa, KS) starting from 1 in 5 then in 2-fold serial dilutions to 1:1,280. The diluted serum samples were incubated for 1 h at 37°C with rAAV-hAAT-*GFP* diluted in an equal volume of DMEM. rAAV vectors were incubated at the same concentration to reach a predetermined final multiplicity of infection (MOI) into a 100-μL final volume for transduction. The optimal transduction for the assay was reached at an MOI of 2,000 vg/cell for AAV-LK03 and AAV-3B and 30,000 vg/cell for AAV8. Purified immunoglobulins (Octagam 10% [v/v]; Octapharma Ltd, Manchester, UK) were used as positive control. Each assay was run in duplicate. The next day, wells were complemented with DMEM and 10% (v/v) FCS. Fluorescence-activated cell sorting BD FACS-Verse™ (BD Bioscience, San Jose, CA) analysis of GFP signal was performed 72 h post-transduction. Samples were considered positive when a 1:5 dilution of serum reduced the vector transduction by 50% or more. The neutralizing antibody titer was determined as the highest positive serum dilution.

### Assay validation

Reproducibility was assessed by inter- and intra-assay coefficients of variation (CV). The inter-assay CV was established by three assays spaced by 2–14 days on four different samples. The intra-assay CV was established by seven repeats on four different samples at a dilution corresponding to the titer for each sample tested. Both ELISA and neutralizing assay showed inter- and intra-assay CV <15%.

### Statistical analysis

Descriptive analysis of human samples used four main age groups: 0–6 months to reflect seroprevalence from maternal antibodies; 7 months to 2 years, age reported to have the lowest seroprevalence^24^; 3–17 years, pediatric age excluding infancy; and ≥18 years, adulthood. Comparisons of continuous variables were performed using the chi-squared test performed in GraphPad Prism 5.0 software (San Diego, CA, USA). Finally *p* values <0.05 were considered statistically significant.

## Results

### Anti-AAV-LK03 IgG and Nab rates in humans vary with age

**Figure 1.**
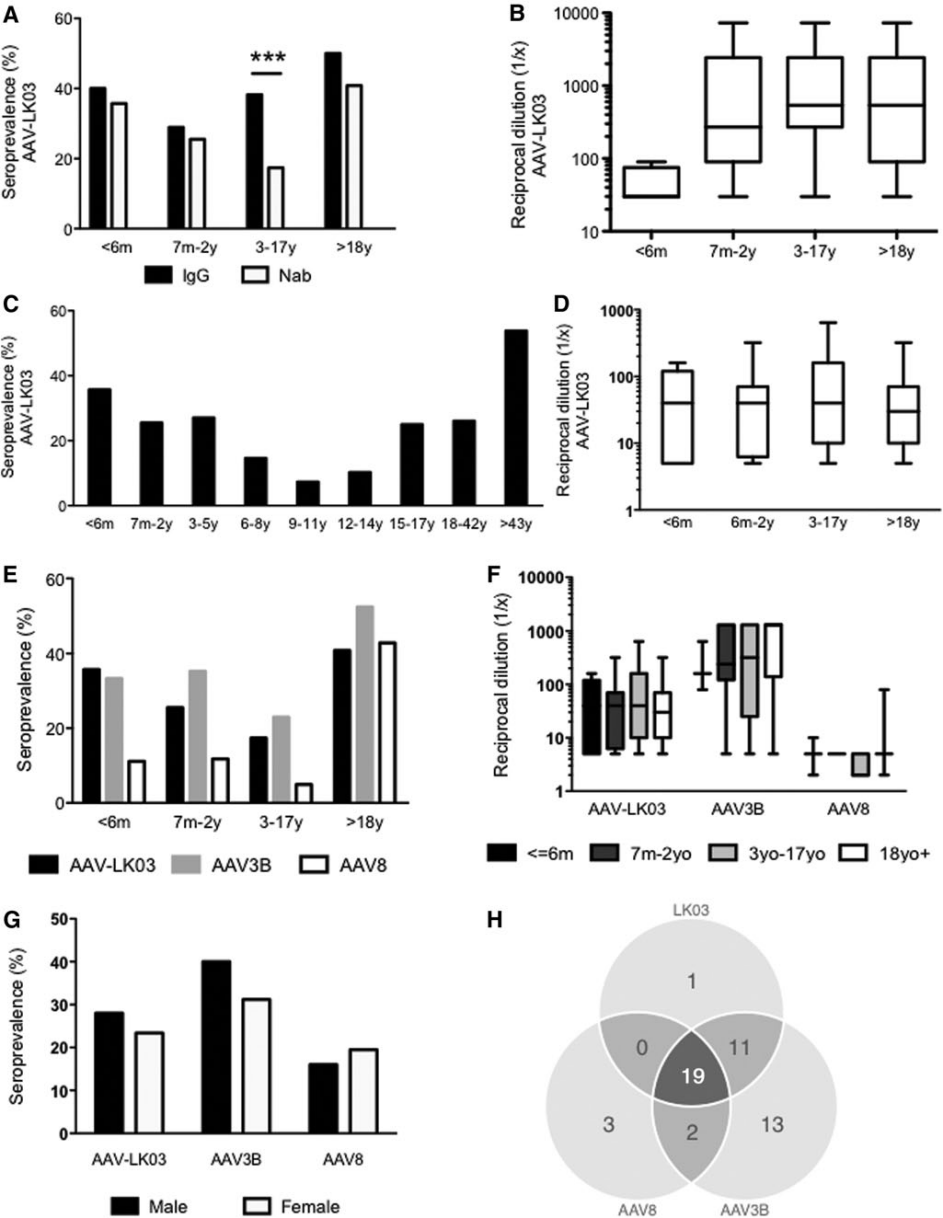
Seroprevalence of immunoglobulin (Ig)G and neutralizing antibodies (Nab) against adeno-associated virus (AAV)-LK03, 3B, and 8 in humans. **(A)** Anti-IgG and Nab seroprevalences against AAV-LK03; **(B)** anti-IgG titers against AAV-LK03; **(C)** Nab seroprevalence against AAV-LK03 according to age; **(D)** Nab titers against AAV-LK03 according to age; **(E)** comparison of Nab seroprevalence against AAV-LK03, 3B, and 8; **(F)** comparison of Nab titers against AAV-LK03, 3B, and 8; **(G)** Nab seroprevalence according to sex; **(H)** cross-reactivity of Nab between serotypes. Vertical bars represent the mean. Box-and-whisker plots represent the minimum, the mean, the maximum, and the 25th and 75th percentiles. Samples: **(A, B)** IgG seroprevalence against AAV-LK03, *n* = 189; **(C–F)** Nab seroprevalence against AAV-LK03, *n* = 323; **(E, F)** against AAV3B and AAV8, *n* = 129; **(G)** male and female samples, *n* = 50 and *n* = 77, respectively; **(H)** *n* = 49.

A random representation of serum samples from neonates to adults was tested for total IgG (*n* = 185) and Nab (*n* = 323) against AAV-LK03 and showed an overall rate of 39% and 23%, respectively. Anti-AAV-LK03 IgG seroprevalence increased from 29% in the age cohort of 7 months to 2 years to 50% in the adult cohort (Fig. 1A). IgG seroprevalence was significantly higher than Nab seroprevalence in the age cohort of 3–17 years (39%, *n* = 89 versus 17%, *n* = 213; *p* < 0.0001). Titers of anti-AAV-LK03 IgG (*n* = 185) in infants younger than 6 months were much lower than in older children (Fig. 1B).

### Lowest Nab rates against AAV-LK03 are observed during late childhood and early adolescence

Among 323 samples tested, 260 were from pediatric patients (80%; 0–18 years old). Overall Nab rates against AAV-LK03 were 27%, 21%, and 18% at 1:5, 1:10, and 1:20 dilutions, respectively. Presence of Nabs at 1:5 dilution was analyzed in more precisely defined age groups (Fig. 1C). The lowest Nab titers were observed in the cohort of 6- to 14-year-olds with a mean of 11% (*n* = 121) (Fig. 1C). The highest Nab rate was observed in late adulthood (43–84 years old, *n* = 26) at 54% (Fig. 1C). The distribution of Nab titers assessed in seropositive samples (*n* = 54) showed no significant difference within groups (Fig. 1D).

### Anti-AAV-LK03 Nab rate is lower than anti-AAV3B but higher than anti-AAV8 rates

Of the 323 samples used to study AAV-LK03 Nab rates, 129 samples were randomly assigned to assess Nab rates against AAV3B and AAV8. At 1:5 dilution, overall Nab seroprevalence was 35% (minimum 23% in 3- to 18-year-olda; maximum 52% in the >18 cohort) and 19% (minimum 5% between 3 and 18 years; maximum 43% when >18 years) for AAV3B and AAV8, respectively. Pediatric Nab seroprevalence rates against AAV-LK03, AAV3B, and AAV8 were 20%, 26%, and 7%, respectively. For all serotypes, the lowest seroprevalence was observed in the age range of 3–17 years (17%, 23%, and 5% for AAV-LK03, AAV3B, and AAV8, respectively) (Fig. 1E). In the pediatric population, AAV8 was consistently the capsid with the lowest Nab rate, whereas in adulthood anti-AAV-LK03 was the least prevalent (Fig. 1E). Nab rates against AAV-LK03 and AAV3B were similar in <6-month-old infants, but in the older cohorts AAV3B consistently exhibited a higher Nab rate (Fig. 1E). When the capsids were considered individually, titers assessed in seropositive samples (AAV-LK03, *n* = 54; AAV3B, *n* = 45; AAV8, *n* = 24) were not substantially different between age groups. However anti-AAV8 antibody titers were 1-log and 1.5-log below those of anti-AAV-LK03 and anti-AAV3B, respectively (Fig. 1F).

### No sex-associated difference in Nab rates

Nab rates were tested in 50 and 77 randomly assigned male and female samples, respectively. No significant difference was observed for AAV-LK03 (*p* = 0.5), AAV3B (*p* = 0.3), and AAV8 (*p* = 0.6) (Fig. 1G).

### Cross-reactivity of Nab between AAV-LK03, AAV3B, and AAV8

In 129 samples analyzed for Nab against the three serotypes, 49 were positive against at least one capsid (38%). In this subset, 19/49 (39%) were cross-reactive against all three serotypes studied (Fig. 1H). Samples with anti-AAV-LK03 Nab were highly cross-reactive against AAV3B, but less against AAV8 (97% and 61%, respectively) (Table 1). When tested reciprocally, samples with anti-AAV3B Nab showed cross-reactivity rates of 67% and 47% with AAV-LK3 and AAV8, respectively (Table 1).

**Table 1.** Cross-reactivity of neutralizing antibodies to AAV-LK03, 3B, and 8 in humans

	*AAV-LK03*	*AAV3B*	*AAV8*
AAV-LK03	—	97%	61%
AAV3B	67%	—	47%
AAV8	79%	87%	—

### Nab rates against AAV-LK03, AAV3B, and AAV8 in nonhuman primates and comparison with humans

**Figure 2.**
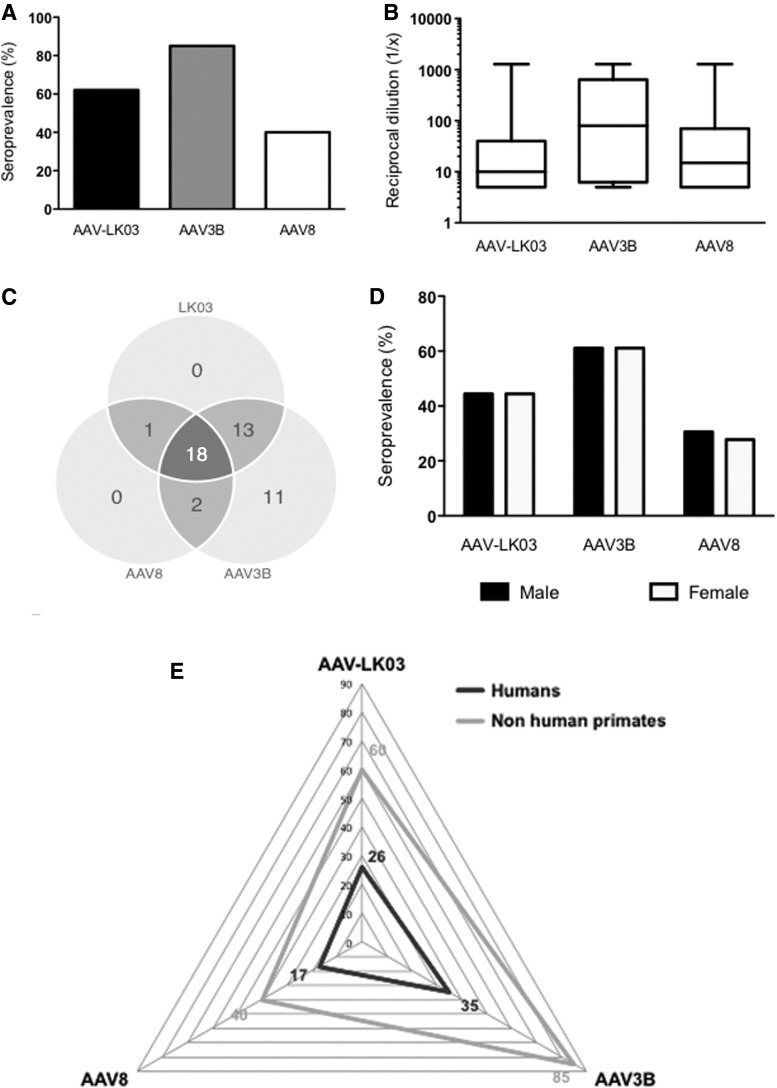
Seroprevalence of Nab against AAV-LK03, 3B, and 8 in juvenile nonhuman primates. **(A)** Comparison of Nab seroprevalence in nonhuman primates and humans; **(B)** Nab titers in nonhuman primates; **(C)** sex comparison; **(D)** cross-reactivity of Nab between serotypes in nonhuman primates; **(E)** comparison of Nab seroprevalence between serotypes in humans and nonhuman primates. Vertical bars represent the mean. Box-and-whisker plots represent the minimum, the mean, the maximum, and the 25th and 75th percentiles. Samples: **(A, E)** macaque samples, *n* = 72; human samples against AAV-LK03, *n* = 323; against AAV3B and AAV8, *n* = 129; **(B, C)** *n* = 72; **(D)** *n* = 45.

Fifty-two samples from juvenile rhesus macaques were tested for Nab at a serum dilution of 1:5. Nab rates against AAV-LK03, AAV3B, and AAV8 were 62%, 85%, and 40%, respectively (Fig. 2A). For individual serotypes, Nab rates did not show any significant difference between humans and nonhuman primates. Titers were not significantly different between capsids (Fig. 2B). The profile of cross-reactivity in macaques was similar to that observed in humans with Nab against AAV-LK03 cross-reacting heavily with AAV3B (97%) but much less with AAV8 (59%). Samples with Nab against AAV8 were highly cross-reactive against AAV-LK03 and AAV3B (90% and 95%, respectively) (Table 2 and Fig. 2C). Nab rates tested according to sex in sex-matched groups (*n* = 26 per sex) showed no significant difference for AAV-LK03 (*p* = 1), AAV3B (*p* = 1), or AAV8 (*p* = 0.8) (Fig. 2D). Nab rates were 2 to 3 times higher in juvenile macaques compared to humans (Fig. 2E).

**Table 2.** Cross-reactivity of neutralizing antibodies against AAV-LK03, 3B, and 8 in nonhuman primates

	*AAV-LK03*	*AAV3B*	*AAV8*
AAV-LK03	—	97%	59%
AAV3B	70%	—	45%
AAV8	90%	95%	—

## Discussion

This study presents the rates of IgG and Nab seroprevalence against selected wild-type and engineered AAV capsids, which are commonly used for liver-directed gene therapy, in large UK-based pediatric and adult cohorts and juvenile nonhuman primates.

In humans, the IgG seroprevalence profile was in accordance with the humoral immune response against AAVs previously reported,^24,41^ with a higher prevalence before 6 months of age, likely caused by passive transfer of maternal IgG *in utero* or during breastfeeding, a minimal rate between 7 months and 2 years after clearance of maternal IgG, followed by a progressive rise during childhood and adolescence to reach a maximal rate in adulthood.

Nab seroprevalence against all three serotypes tested was lower than previously reported. Anti-AAV-LK03 Nab seroprevalence overall was 23% in our population (containing 80% pediatric samples) and 54% in late adulthood. Anti-AAV-LK03 Nab seroprevalence rates of 67% and 91% were reported in recent studies in European (*n* = 21, cutoff 1:5 dilution)^39^ and Chinese (*n* = 100, cutoff 1:20 dilution)^38^ adult populations, respectively. Pediatric anti-AAV3B Nab seroprevalence was 26%, which is also lower than 44% reported previously in a pediatric population from the United States.^42^ Similarly, the pediatric anti-AAV8 Nab seroprevalence rate of 7% found here is lower than previous findings in the U.S. pediatric population, with 16% and 23% reported in 752^24^ and 62^25^ pediatric samples (cutoff 1:5 dilution). In our study, anti-AAV8 Nab seroprevalence in adults was found to be 18%, which falls in the low range of previous reports summarized by Jeune *et al.*^30^ Surprisingly, detailed testing of anti-AAV-LK03 Nab seroprevalence rates across age ranges showed a higher seroprevalence rate before 6 months of age, followed by a gradual reduction in seroprevalence reaching a minimum of 7%–10% between 6 and 14 years of age, before increasing progressively during adolescence and adulthood. A similar tendency was observed for serotypes AAV3B and AAV8. This finding is in contrast with some reports for AAV2 and AAV8 serotypes that observed minimal seroprevalence rates between 7 and 11 months followed by a progressive increase, although variations in seroprevalence in childhood have been observed.^24^ We hypothesize that recurrent viral infections in early childhood (AAV and other AAV helper viruses like herpes simples virus 1 and adenoviruses) facilitated by socialization in nurseries or preschools trigger a transiently higher Nab seroprevalence, which later decreases with reduced exposure to these helper viruses in late childhood. More longitudinal pediatric studies are required to rigorously test this hypothesis. This loss of the neutralizing effect of anti-AAV antibodies has previously been observed with wild-type capsids in pediatric patients with hemophilia A.^25^ Five children with Nab against AAV2 (*n* = 1), AAV5 (*n* = 3), and AAV8 (*n* = 1) showed loss of IgG neutralizing effect when monitored prospectively over 4 years. Underlying immunological mechanisms are not well understood. Antiviral serologic memory is a complex phenomenon with peripheral memory B cells and antibody-secreting plasma cells, which might be independently regulated.^43^

Various factors influence Nab prevalence, such as geographic location, living conditions, population density, health care system, and genetic background.^44^ The decreased Nab seroprevalence in late childhood and adolescence favors application of AAV gene therapy compared to adults. This knowledge is of paramount interest for the design of clinical trials testing AAV gene therapy products targeting the liver, especially for inherited metabolic liver diseases because teenagers are at an increased risk of mortality (*e.g.*, in urea cycle defects).^45^ Moreover, liver growth slows substantially by the age of 12 years, and therapeutic transgene expression from AAV episomes is therefore likely to be more durable than in early childhood.^22^ Finally, the fact that the neutralizing effect of anti-AAV antibodies could diminish over time supports the need for further (*e.g.*, yearly) Nab screening of children who might benefit from an ongoing trial of AAV gene therapy.

Compared to its close parental wild-type capsid AAV3B, AAV-LK03 showed a lower Nab seroprevalence (23% versus 35%) and lower titers (1/40 versus 1/200). This finding is consistent with reduced neutralizing effect observed with pooled intravenous immunoglobulins with AAV-LK03 compared to AAV3B.^35,39^ Samples with anti-AAV-LK03 Nab showed cross-reactivity rates of 97% and 63% AAV3B and AAV8, respectively. This finding suggests that patients may not benefit from the use of these capsids if they are selected as vectors for secondary reinjection after the initial treatment with AAV-LK03.

AAV5-derived vector has been successfully used in clinical trial for hemophilia A^5^ but did not demonstrate clinical benefit for acute intermittent porphyria.^46^ Although AAV5 presents up to 1-log reduced transduction efficacy compared with AAV8,^47^ this capsid has shown low seroprevalence in humans^30^; however, high cross-reactivity rates of 55%^25^ and 89%^26^ have been reported with AAV8, making this capsid unlikely to be suitable for re-injection.

In conclusion, high cross-reactivity of Nab between AAV-LK03, AAV3B, and AAV8 makes a re-injection between these serotypes unlikely to achieve significant transduction. Overall anti-AAV-LK03 Nab seroprevalence is low (23%), particularly in late childhood, which makes this age group particularly suitable for AAV gene therapy. Nab seroprevalence also decreases steadily during the first decade, suggesting that Nab-positive pediatric patients excluded from AAV gene therapy clinical trials could be rescreened for eligibility because this status may change. Larger longitudinal prospective studies will be needed to confirm our observation.
